# Design and Characterization of Hybrid Glucose-Powered Enzymatic Biofuel Cells Based on the Combination of Different Gold-Based Nanocompounds

**DOI:** 10.1021/acs.langmuir.6c02417

**Published:** 2026-07-04

**Authors:** Natalija German, Almira Ramanaviciene, Arunas Ramanavicius

**Affiliations:** † Department of Immunology and Bioelectrochemistry, State Research Institute Centre for Innovative Medicine, Santariskiu 5, LT-08406 Vilnius, Lithuania; ‡ NanoTechnas − Center of Nanotechnology and Materials Science, Faculty of Chemistry and Geosciences, 54694 Vilnius University, LT-03225 Vilnius, Lithuania; § Department of Nanotechnology, State Research Institute Center for Physical Sciences and Technology (FTMC), Sauletekio Ave. 3, LT-10257 Vilnius, Lithuania

## Abstract

This study highlights the development of 5 different membraneless glucose enzymatic biofuel cells, which are capable of operating in dual modes (biofuel cells and sensors). In these biofuel cells, different bioanodes were used, consisting of graphite rods (GRs) modified with gold nanoparticles (AuNPs), dendritic gold nanostructures (DAuNSs), or both types of above-mentioned nanostructures covered by cystamine (Cys), a redox mediator (1,10-phenathroline-5,6-dione (PD)), and differently immobilized glucose oxidase (GOx). The Cys-based self-assembled monolayer (SAM) provided a platform for covalent GOx immobilization on the surface of the above-mentioned gold-based structures. A single-compartment-based membraneless design was applied for all three here designed hybrid enzymatic biofuel cells (G-EBFCs), which were all powered by glucose. Electrically conducting nanocompounds served as support for immobilization, while PD served as a redox mediator to enhance the current of G-EBFCs. The electrochemical behavior of the fabricated bioanodes, assessed by G-EBFCs, was investigated by cyclic voltammetry (CV) and direct voltage measurements. Although both GR/Cys/AuNPs/PD/GOx and GR/AuNPs/Cys/PD/GOx bioanodes were characterized by rather high power density, surface concentration of GOx, sensitivity, and low limit of detection, the GR/AuNPs/Cys/PD/GOx bioanode was considered more suitable for glucose biosensing in real samples due to its long-term stability and excellent anti-interference capability. The technological challenges discussed in this study open new horizons for simpler and more cost-effective designs of hybrid G-EBFCs and glucose biosensors, suitable for biomedical applications and the monitoring of beverage quality.

## Graphical abstract

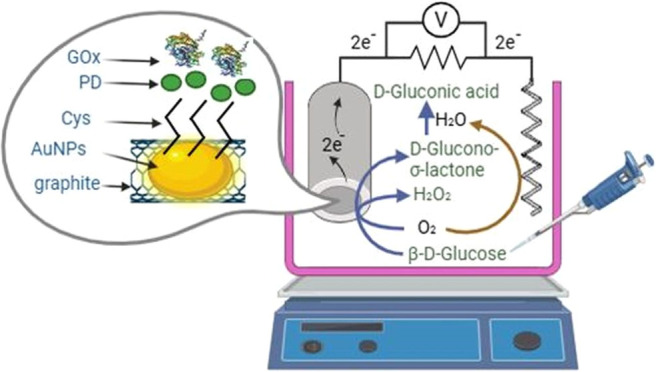

## Introduction

Biofuel cells (BFCs) are bioelectrochemical devices that have attracted considerable attention over the past few decades because of their potential as alternative, economical, and renewable energy sources and their possible application in some self-powered biosensors, brain neurostimulators, gastric electrical stimulators, and pacemakers.^1–4^ BFCs are energy conversion devices that can efficiently convert the chemical energy of biofuels (glucose, fructose, alcohol, or hydrogen) into electrical energy through biocatalytic processes.^3–5^ Glucose and oxygen (O_2_) are ideal energy sources because they are readily available in the environment and are continuously produced during metabolism,^1,5^ and they are broadly available in biological fluids.^4^ Therefore, glucose-powered BFCs can be employed as implantable biosensors to monitor the glucose level in the blood of patients with diabetes mellitus, which is considered one of the most common causes of death and cardiovascular diseases, retinal damage, kidney failure, blindness, and limb amputation worldwide.^6,7^ Approximately 20 million people globally are affected by type 1 diabetes mellitus, which is most commonly diagnosed during childhood or early adulthood.^6^

In the past few decades, the number of studies and publications on microbial biofuel cells,^8–11^ glucose-powered enzymatic biofuel cells (G-EBFCs),^12–15^ and hybrid G-EBFCs^16–25^ has increased, indicating a great interest in the field of biofuel cell development.^4^ The power in G-EBFCs or self-powered glucose sensors is generated by the oxidation of glucose with glucose oxidase (GOx) at the surface of a bioanode (glucose → gluconolactone + 2H^+^ + 2e^–^) and the reduction of oxygen to water (H_2_O) at the surface of a cathode (1/2O_2_ + 2H^+^ + 2e^–^ → H_2_O).^5,10,16^ The defining characteristic of BFCs or hybrid BFCs is enzymatically catalyzed electron release at the bioanode, coupled with subsequent electron consumption at the cathode.^10–16^ The membraneless design of hybrid G-EBFCs increases the oxygen supply in the cell, thereby enhancing the oxygen reduction reaction, which governs the overall performance of the biofuel cell.^15,21^ The power generated during the enzymatic reaction can be used to operate bioelectronic^13–16^ and medical^4^ devices. Unlike widely investigated G-EBFCs, hybrid G-EBFCs exhibit high stability over extended periods, which may play a significant role in the development of implantable medical devices^5^ and self-powered biosensors.^20^ Hybrid G-EBFCs are considered a low-cost alternative to abiotic and enzymatic biofuel cells.^23–25^

Enzymes are widely used in the design of bioreactors, implantable medical devices, and environmental monitoring systems due to their high specific activity.^12,13^ Such an enzyme, like glucose oxidase, characterized by high catalytic activity, stability, and specificity to glucose oxidation,^4^ is a naturally occurring high-molecular-weight protein with an active center deeply embedded within the protein shell, which limits electron transfer between the enzyme and electrode.^3,12^ This challenge can be solved through both direct electron transfer^26^ or mediated electron transfer.^12,21^ Electron transfer mediators may be employed to enhance the efficiency of electron transfer between an enzyme and a bioanode, as well as the current output and power density (*J*) of BFCs.^5,10^ This might involve the use of redox-active compounds, such as 2,6-pyridinedicarboxylic acid,^19^ p-benzoquinone,^22^ ferritin (Frt),^17^ ferrocene-methanol (Fc-MeOH),^24^ and 1,10-phenathroline-5,6-dione (PD).^27,28^

Nanotechnology has opened new horizons for improving the performance of G-EBFCs^13,28^ by making them powerful implantable medical devices.^4^ The modification of bioelectrodes with nanocompounds such as nanoporous gold (NPG),^14^ gold nanoparticles (AuNPs),^27,29^ dendritic gold nanostructures (DAuNSs),^30^ palladium nanoparticles (PdNPs),^20^ platinum nanoparticles (PtNPs),^21,29^ maghemite nanoparticle (γ-Fe_2_O_3_NP),^21^ or multiwalled carbon nanotubes (MWCNTs),^15,20,23,31^ is able to improve the performance of enzyme-based bioelectrodes by increasing the surface area,^5,17,18^ enhancing electron transfer,^3,21^ improving catalytic properties, and optimizing electrode design.^4^ The performance of abiotic glucose BFCs based on metal catalysts depends on the properties of the catalyst, the concentration, pH, and functional conditions of the designed BFCs.^29^

Usually, the nature of the anode and cathode, and the stability of the biocatalyst affect the power generated by BFCs.^23^ Graphite rod (GR) is considered the most versatile and simplest anode material due to its large surface area, cost-effectiveness, ease of use, high conductivity, biocompatibility, and chemical stability.^9,11^ Bandapati et al. demonstrated the performance (low price, rapid surface renewal, simplicity of modification, and miniaturization) of the bioanode–pencil graphite (PG) electrode modified with carboxylic acid-functionalized multiwalled carbon nanotubes (COOH-MWCNTs) and with immobilized GOx (PG/COOH-MWCNTs/GOx) for glucose oxidation.^22^ Platinum (Pt) catalysts are usually used as cathodes in BFCs to increase the rate of O_2_ reduction and are employed for the construction of long-term implantable medical devices.^5,16,32^ Kloke et al. demonstrated the performance (a power density of 5.1 μW cm^–2^) of an implantable long-term glucose BFCs based on porous Pt electrodes electrodeposited by platinum–copper alloy.^32^ In BFCs, the voltage or electrode potentials are typically measured using low-cost multimeters.^9,19^ Cystamine (Cys) is typically used to functionalize and stabilize the gold surface due to the formation of a self-assembled monolayer (SAM) through gold–thiol (Au–S) bonds.^33–35^ The SAM improves the immobilization of biomolecules by enhancing the surface area of the electrode^33,34^ and the stability of biological matrices.^35^

Most reported glucose BFCs are effective but costly due to the use of expensive reagents and electrodes,^19,32^ difficulty of construction,^25^ as well as their limited stability.^14,23,31^ A complex and expensive hybrid G-EBFC was manufactured based on a gold (Au) electrode modified with MWCNTs, electroplated with palladium (Pd_plate_), immobilized with a mixture of GOx, poly­(3-anilineboronic acid) (PABA), and PdNPs, and covered with chitosan (CS) film (Au/MWCNTs/Pd_plate_/GOx-PABA-PdNPs/CS) as a bioanode. The Au/MWCNTs/Pt cathode exhibited a high power density of 54.5 μW cm^–2^ and a short-circuit current of 1.25 mA cm^–2^.^20^ The hybrid G-EBFC consisted of (i) a bioanode based on a glassy carbon (GC) electrode modified with ''Vulcan'' carbon (VC) and γ-Fe_2_O_3_NP, and immobilized with GOx (GC/VC/γ-Fe_2_O_3_NP/GOx), and (ii) a cathode based on a mixture of PtNPs and VC, characterized by a high power density of 30 μW cm^–2^, but a short lifetime (2 days).^21^ Torigoe et al. constructed a high power (58.2 mW cm^–2^), but short-term stable (3 h (0.125 days)) abiotic glucose fuel cell based on VC cloth/AuNPs-PtNPs anode catalyst.^29^ This has encouraged the search for alternative electrode designs to solve challenges such as low power density, power current, and poor operational stability.^3^

The ultimate goal of this research is to develop effective, simple, low-cost self-powered membraneless hybrid G-EBFCs by combining gold nanocompounds and SAM to evaluate their performance and to explore potential applications in the physiological environment for glucose biosensing. This work demonstrates the advantages of graphite-based electrodes in designing cost-effective bioanodes for bioelectrocatalytic applications.

## Experimental Section

### Materials

Glucose oxidase (type VII, from *Aspergillus niger*, 208 units mg^–1^ protein), cystamine dihydrochloride, and l-ascorbic acid (AA, C_6_H_8_O_6_) were supplied by Fluka Chemie GmbH (Buchs, Switzerland). Monosaccharides (D-(+)-glucose, D(−)-fructose, D­(+)-mannose, D­(+)-galactose (C_6_H_12_O_6_), and D­(+)-xylose (C_5_H_10_O_5_)), disaccharide (D­(+)-saccharose (C_12_H_22_O_11_)), tannic acid, and hydrochloric acid were obtained from Carl Roth GmbH + Co. (Karlsruhe, Germany). Tetrachloroauric acid trihydrate, 1,10-phenathroline-5,6-dione, graphite rod (diameter of 3 mm), 96% ethanol, and human serum (Type AB) were purchased from Sigma-Aldrich (Saint Louis, MO, USA). Trisodium citrate dihydrate and potassium chloride (KCl) were obtained from Penta (Praha, Czech Republic). Uric acid (UA, C_5_H_4_N_4_O_3_) and 25% solution of glutaraldehyde (GA) were obtained from AppliChem GmbH (Darmstadt, Germany); potassium nitrate was obtained from Acros Organics (Morris Plains, NJ, USA), potassium hydroxide was obtained from Merck KGaA (Darmstadt, Germany); hexaammineruthenium­(III) chloride (Ru­(NH_3_)_6_Cl_3_) was obtained from Fisher Scientific (Waltham, MA, USA), and α aluminum oxide (α-Al_2_O_3_, 0.3 μm, type N) was obtained from Electron Microscopy Sciences (Hatfield, MA, USA). All chemicals employed in these studies were of analytical grade. Deionized water was used in all experiments. Saccharide solutions were prepared 24 h prior to the experiments to ensure complete mutarotation. The sodium acetate (SA) buffer (pH 6.0), containing 0.05 mol L^–1^ sodium acetate trihydrate (Reanal, Budapest, Hungary) and 0.1 mol L^–1^ KCl, was employed as the electrolyte and dilution solution, respectively. To prepare 38 mmol L^–1^ PD, 96% solution of ethanol was used. Red wine “Montmeyrac” was purchased from Les Grands Chais de France (Landiras, France); coconut and almond milk “Alpro” was obtained from Issenheim (France); apple juice “Kubuś” was obtained from Maspex (Olsztynek, Poland), and mandarin juice “Elmenhorster” was obtained from Eckes-Granini Baltic (Kaisiadorys, Lithuania).

### Synthesis of Gold Nanoparticles and Dendritic Gold Nanostructures, and the Fabrication of Bioanodes

The 13 nm AuNPs (50 μg mL^–1^) (Figure S1) were prepared according to our previously described procedure,^27^ which is provided in the Supporting Information. The electrochemical synthesis of long, thin, and branched DAuNSs (Figure S2) was performed according to the methodology presented previously in a research study,^30^ and is provided in more detail in Supporting Information.

The detailed design of the membraneless hybrid G-EBFCs construction and the schematic reaction of glucose oxidation are presented in Figure 1. First, to construct the (i) GR/Cys/AuNPs/PD/GOx, (ii) GR/AuNPs/Cys/PD/GOx, (iii) GR/DAuNSs/Cys/AuNPs/PD/GOx, (iv) GR/DAuNSs/Cys/PD/GOx, and (v) GR/Cys/PD/GOx bioanodes, graphite rods with a geometrical surface area of 0.071 cm^2^ were polished using fine emery paper, followed by polishing with wet α-Al_2_O_3_ powder, rinsed with deionized water, dried at room temperature (+20 ± 2 °C), and subsequently sealed into a silicone tube. No electrochemical activation was applied to the GR surface.

i. To fabricate the GR/Cys/AuNPs/PD/GOx electrode, GR was kept in 5.0 mmol L^–1^ Cys at room temperature for 16 h. Then, the GR/Cys electrode was washed with deionized water, dried, and covered with 3 μL of colloidal solution containing 13 nm AuNPs (50 μg mL^–1^). Then, 3 μL of 25 mg mL^–1^ GOx was deposited using a drop-coating method on the dried GR/Cys/AuNPs electrode. After the evaporation of the solvent (water), 4.5 μL of 38 mmol L^–1^ PD solution was distributed on the surface of the electrode and then dried in air. Later on, the GR/Cys/AuNPs/PD/GOx assembly was stored for 15 min in a 25% GA solution at room temperature to cross-link the biomolecule (GOx) and Cys amine groups.^34,36^ Finally, the modified working electrode was rinsed with deionized water to remove unbound GOx and dried at room temperature.

**Figure 1:**
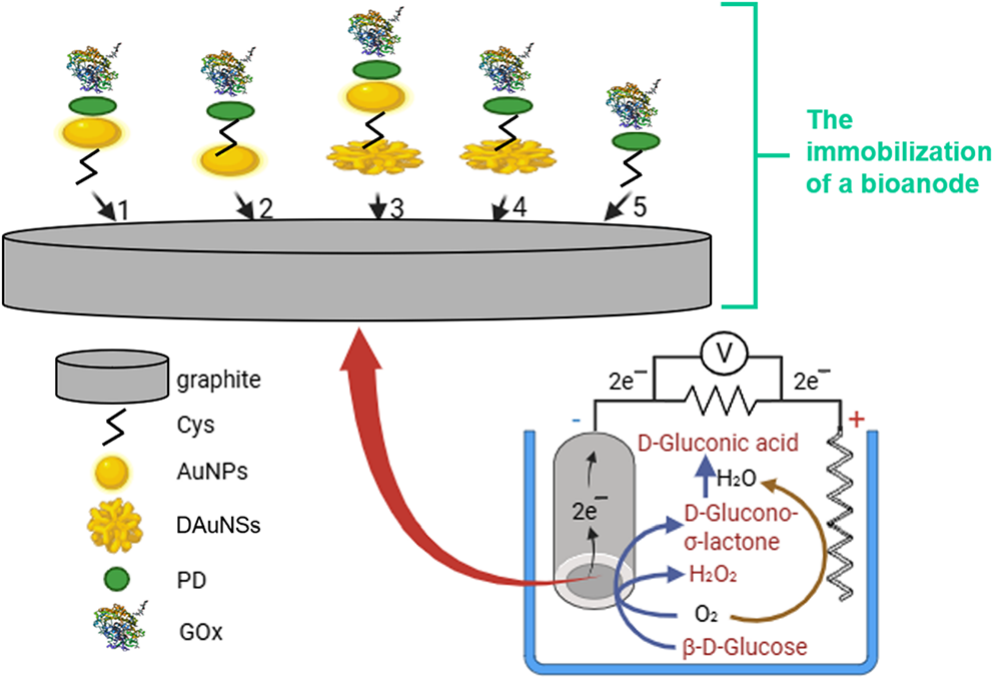
Schematic representation of membraneless hybrid G-EBFCs based on graphite rods modified with Cys/AuNPs/PD/GOx (**1**), AuNPs/Cys/PD/GOx (**2**), DAuNSs/Cys/AuNPs/PD/GOx (**3**), DAuNSs/Cys/PD/GOx (**4**), and Cys/PD/GOx (**5**).

ii. The GR/AuNPs/Cys/PD/GOx electrode was prepared following the methodology described above, with the only difference being the order of modification procedures. The cystamine and gold nanoparticle coating steps were performed in a different order: the surface of the GR was initially coated with 13 nm AuNPs and then subsequently modified with Cys to form a Cys-based self-assembled monolayer.
iii. To design the GR/DAuNSs/Cys/AuNPs/PD/GOx electrode, GR was initially modified with electrochemically synthesized dendritic gold nanostructures according to the procedure described in previously reported research,^30^ and the detailed description is presented in the Supporting Information. The subsequent modification steps were carried out following the same procedure as described above for the fabrication of the previously described GR/Cys/AuNPs/PD/GOx electrode.
iv. The GR/DAuNSs/Cys/PD/GOx electrode was prepared using the same methodology as for the (iii) GR/DAuNSs/Cys/AuNPs/PD/GOx electrode, without incorporating 13 nm AuNPs.
v. The GR/Cys/PD/GOx electrode was designed without the deposited 13 nm AuNPs or electrochemically synthesized DAuNSs and was used as a control electrode.

Cys is expected to bind to modified gold-based nanocompounds (AuNPs or DAuNSs) modified GR electrodes via the formation of Au–S bonds, which is consistent with the well-known chemistry of thiol-containing molecules on gold surfaces. In contrast, the adsorption of Cys on the bare GR electrode does not rely on the formation of Au–S bonds and is therefore attributed to weaker interactions, including physisorption, hydrogen bonds with oxygen-containing surface functional groups naturally present in the graphite structure, and interactions with surface defect sites.

### Electrochemical and Statistical Assessment of the Designed Electrochemical Systems

The above-described differently designed bioanodes (GR/Cys/AuNPs/PD/GOx, GR/AuNPs/Cys/PD/GOx, GR/DAuNSs/Cys/AuNPs/PD/GOx, GR/DAuNSs/Cys/PD/GOx, and Cys/PD/GOx) were implemented into G-EBFCs. A positively charged counter Pt spiral (−2 cm^2^), purchased from BASi Research Products (West Lafayette, IN, USA), was used as a cathode of the G-EBFCs.

A resistor set was used to imitate the external load, and a digital multimeter UT58B from UNI-Trend (Hong Kong, Guangdong province, China) was used to measure the voltage (*E*) and current (*I*) generated by the G-EBFCs. Bioanodes and cathodes in each single-compartment-based G- EBFCs were located at a constant distance of 1 cm. All investigations were performed in a 0.05 mol L^–1^ SA buffer (pH 6.0).

As shown in Figure 1, β-d-glucose in the presence of oxygen is oxidized by the enzyme (GOx) into d-glucono-δ-lactone with subsequent transformation to d-gluconic acid, while O_2_ is reduced to hydrogen peroxide (H_2_O_2_), which is finally converted into H_2_O.^2,37,38^ During enzymatic reactions, electrons from organic substances are accepted by GOx, which are transferred to the anode of G-EBFCs, generating an external current, while protons are transferred through the solution toward the cathode, where they are involved in the reduction of dissolved oxygen to water.^8,9^ Thus, O_2_ acts as an additional electron acceptor for GOx,^32^ which catalyzes the oxidation of glucose.^21^ The application of gold nanocompounds and PD is expected to enhance electron transfer from the GOx redox center to the electrode.^27^

The influence of an external load for all five hybrid G-EBFCs based on the GR/Cys/AuNPs/PD/GOx, GR/AuNPs/Cys/PD/GOx, GR/DAuNSs/Cys/AuNPs/PD/GOx, GR/DAuNSs/Cys/PD/GOx, and Cys/PD/GOx electrodes, respectively, was tested using externally connected resistors with external resistances of 0.098, 0.985, 5.03, 9.80, 49.0, 97.0, 512, 2190, 3810, and 4730 kΩ in the absence and presence of 40.3 mmol L^–1^ glucose. The power density, characterized as a power generated per unit of surface, was calculated using the equation^9^

1 $$J={PA}^{-1}=E^{2}\cdot {\left(RA\right)}^{-1}$$

where *J* is the power density (W cm^–2^), *P* is the power output (W), *A* is the surface area of the GR (m^2^), *E* is the voltage (V), and *R* is the resistance (Ω).

The voltage for hybrid G-EBFCs based on the GR/Cys/AuNPs/PD/GOx, GR/AuNPs/Cys/PD/GOx, and GR/DAuNSs/Cys/AuNPs/PD/GOx bioanodes in the presence of 40.3 mmol L^–1^ glucose using a 49.0 kΩ resistor was measured. The value of the current was calculated using Ohm’s law (*I* = *ER* ^*–*1^) according to previously reported research.^10^

The computerized potentiostat/galvanostat Autolab/PGSTAT 302N (EcoChemie, Utrecht, The Netherlands), controlled by GPES 4.9 software (AUT83239), was connected to a three-electrode system. This system comprised the corresponding bioanode as the working electrode, a 2 cm^2^ Pt spiral as an auxiliary electrode, and Ag/AgCl_(3 mol L^–1^_ _KCl)_ from Metrhom (Herisau, Switzerland) as the reference electrode, and was used to investigate the electrochemical characteristics of bioanodes. The bioanodes were assessed by cyclic voltammetry (CV) in an unstirred 0.05 mol L^–1^ SA buffer (pH 6.0). A potential range from −0.80 to +0.80 V vs. Ag/AgCl_(3 mol L^–1^_ _KCl)_, a step potential of 0.0024 V, and a potential sweep rate (*v*) of 0.05 V s^–1^ were used for CV-based investigation.

All electrochemical measurements were conducted at least three times, and the measured results were statistically analyzed and presented as mean values with corresponding error bars. The evaluation of the intercept, the slope, and the determination coefficient (*R* ^2^) of the calibration curve was determined by SigmaPlot 13 software (Systat Software Inc., San Jose, CA, USA, demo version). The limit of detection (LOD), the lowest concentration that can be detected statistically significantly, was estimated using the linear range (LR), the slope angle, and three standard deviations. To estimate the sensitivity of the created hybrid G-EBFCs, the slope of the linear plot was related to the surface area of the graphite electrode.

### Estimation of the Surface Coverage of Bioanodes by GOx

For the estimation of the surface coverage of bioanodes by immobilized GOx, the peak current is directly proportional to the quantity of electroactive enzyme molecules on the electrode. To evaluate the surface concentration of immobilized GOx (Γ), cyclic voltammograms were registered using the GR/Cys/AuNPs/PD/GOx, GR/AuNPs/Cys/PD/GOx, and GR/DAuNSs/Cys/AuNPs/PD/GOx electrodes in the SA buffer solution (pH 6.0) by sweeping electrode potentials from −0.80 to +0.80 V vs. Ag/AgCl_(3 mol L^–1^_ _KCl)_ at several potential sweep rates of 0.150 to 0.010 V s^–1^. The surface concentration of GOx was estimated using the Brown–Anson equation^39^ while taking into the account the maximum peak current (*I*_p_) registered by cyclic voltammetry.

2 $$I_{p}=n^{2}\cdot F^{2}S_{\mathrm{EASA}}\cdot \Gamma v{\left(4\mathrm{RT}\right)}^{-1}$$

where *I*_p_ is the maximum peak current (A), *n* is the number of electrons transferred during the enzymatic reaction (*n* = 2e^–^ for the GOx-catalyzed reaction), *F* is the Faraday constant (96,485 C mol^–1^), *S*_EASA_ is the electroactive surface area of the modified bioanodes (cm^2^), *R* is the ideal gas constant (8.314 J (mol K)^−1^), and *T* is the temperature (K).

To evaluate the *S*_EASA_ of the GR/Cys/AuNPs/PD/GOx, GR/AuNPs/Cys/PD/GOx, and GR/DAuNSs/Cys/AuNPs/PD/GOx bioanodes, electrochemical investigations were performed in a solution containing 1.0 mmol L^–1^ Ru­(NH_3_)_6_Cl_3_ and 0.1 mol L^–1^ KCl by CV mode using a potential diapason from −0.70 to 0.0 V vs. Ag/AgCl_(3 mol L^–1^_ _KCl)_ and potential sweep rates from 0.175 to 0.050 V s^–1^. The Randles–Sevcik equation^40,41^ was used to calculate the *S*_EASA_ values for modified bioanodes

3 $$I_{p}=2.69\times 10^{5}\cdot n^{3/2}\cdot S_{\mathrm{EASA}}\cdot D^{1/2}\cdot C\cdot v^{1/2}$$

where *n* is the number of electrons (*n* = 1) transferred in the redox reaction (Ru­(NH_3_)_6_^3+^ + e^–^ ⇄ Ru­(NH_3_)_6_^2+^),^40^ *D* is the diffusion coefficient (9.0 × 10^–6^ cm^2^ s^–1^),^40^ and *C* is the concentration of the electroactive species (0.000001 mol·cm^–3^).

### Assessment of Hybrid G-EBFC Stability and Practical Uses

The GR/DAuNSs/Cys/AuNPs/PD/GOx, GR/Cys/AuNPs/PD/GOx, and GR/AuNPs/Cys/PD/GOx bioanodes were stored in SA buffer (pH 6.0) in refrigeration for 21, 38, and 54 days, respectively, to evaluate the stability of the hybrid G-EBFCs. During this period, the change in the voltage was measured using a digital multimeter with a resistor of 49 kΩ connected as an external circuit. Then, the value of the change of voltage was converted to the corresponding current difference. After each measurement, the bioanodes were rinsed with deionized water, dried, and stored in a refrigerator until the next use.

The applicability of the GR/AuNPs/Cys/PD/GOx bioanode in glucose biosensor design and its selectivity toward interfering and electroactive species were investigated using samples of 10-fold diluted human serum and saliva or 100-fold diluted wine, coconut and almond milk, as well as apple and mandarin juices. Measurements were carried out using a digital multimeter with a resistor of 49 kΩ, following the methodology described in our previous references,^42,43^ and a detailed description is presented in the Supporting Information. The detection of glucose in each real sample was carried out using the ‘standard addition’ method.

## Results and Discussion

### Cyclic Voltammograms of Differently Modified Bioanodes

The shape of the cyclic voltammograms and oxidation/reduction peak shifts provides data about some properties of redox reactions and the electron transfer rates.^44^ The electroactivities of the GR/Cys, GR/Cys/AuNPs/PD, GR/Cys/PD, GR/AuNPs/Cys/PD, GR/DAuNSs/Cys/PD, and GR/DAuNSs/Cys/AuNPs/PD electrodes (Figure 2A), as well as the GR/AuNPs/Cys/PD/GOx, GR/Cys/AuNPs/PD/GOx, GR/Cys/PD/GOx, GR/DAuNSs/Cys/PD/GOx, and GR/DAuNSs/Cys/AuNPs/PD/GOx electrodes (Figure 2B) were estimated by cyclic voltammetry according to the methodology presented in the Experimental Section.

**Figure 2:**
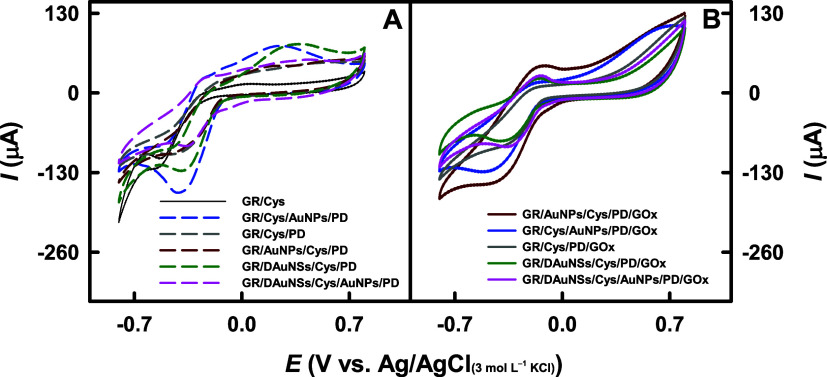
Cyclic voltammograms of the GR/Cys, GR/Cys/AuNPs/PD, GR/Cys/PD, GR/AuNPs/Cys/PD, GR/DAuNSs/Cys/PD, GR/DAuNSs/Cys/AuNPs/PD electrodes without GOx (A) and cyclic voltammograms of the GR/AuNPs/Cys/PD/GOx, GR/Cys/AuNPs/PD/GOx, GR/Cys/PD/GOx, GR/DAuNSs/Cys/PD/GOx, GR/DAuNSs/Cys/AuNPs/PD/GOx electrodes immobilized with GOx (B). Cyclic voltammograms were registered in 0.05 mol L^–1^ SA buffer (pH 6.0) at 0.05 V s^–1^. The GR/Cys electrode exhibits narrow, smooth cyclic voltammograms without any peaks, suggesting the absence of electrocatalytic activity (A). Sufficient redox currents were registered for the electrodes modified with PD, namely the GR/Cys/AuNPs/PD, GR/Cys/PD, GR/AuNPs/Cys/PD, GR/DAuNSs/Cys/PD, and GR/DAuNSs/Cys/AuNPs/PD electrodes (A), with wide, irregular PD redox peaks.

The electrocatalytic activity was determined for the electrodes immobilized with GOx (GR/AuNPs/Cys/PD/GOx, GR/Cys/AuNPs/PD/GOx, GR/Cys/PD/GOx, GR/DAuNSs/Cys/PD/GOx, and GR/DAuNSs/Cys/AuNPs/PD/GOx). As shown in Figure 2B, the GR/AuNPs/Cys/PD/GOx, GR/Cys/AuNPs/PD/GOx, GR/Cys/PD/GOx, GR/DAuNSs/Cys/PD/GOx, and GR/DAuNSs/Cys/AuNPs/PD/GOx electrodes are characterized by rather regular and narrow-shaped cyclic voltammograms with clearly defined anodic and cathodic peaks, which indicate that PD is acting as an electron transfer mediator between the GOx redox center and electrically conducting parts of the electrode.^24^ The cyclic voltammograms recorded by the GR/AuNPs/Cys/PD/GOx, GR/Cys/AuNPs/PD/GOx, GR/DAuNSs/Cys/PD/GOx, and GR/DAuNSs/Cys/AuNPs/PD/GOx electrodes are characterized by increased anodic and cathodic peaks, which can be attributed to the successful modification of the electrodes with biocatalysts (GOx), electrically conducting nanocompounds (AuNPs or DAuNSs), and redox mediators (PD), which facilitates the charge transfer process.

### Assessment of the Performance of Hybrid G-EBFCs

The resistance of the applied external load has a significant impact on the power density of a biofuel cell.^9^ To assess the performance of the hybrid G-EBFCs, the influence of external load resistance on the power density of the GR/Cys/AuNPs/PD/GOx, GR/AuNPs/Cys/PD/GOx, GR/DAuNSs/Cys/AuNPs/PD/GOx, GR/DAuNSs/Cys/PD/GOx, and GR/Cys/PD/GOx bioanodes (Figure 3A) was evaluated according to the procedure described in the Experimental Section.

**Figure 3:**
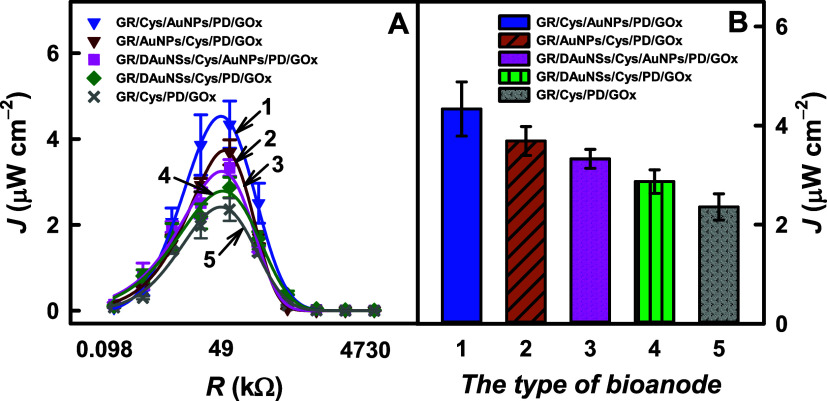
Influence of applied external load (externally connected resistor) on the power density of the hybrid G-EBFCs based on different bioanodes (A), and the maximal power density of the hybrid G-EBFCs based on different bioanodes (B). All measurements were performed in 0.05 mol L^–1^ SA buffer (pH 6.0) in the absence and presence of 40.3 mmol L^–1^ glucose. A,B - GR/Cys/AuNPs/PD/GOx (1, blue line/column), GR/AuNPs/Cys/PD/GOx (2, brown line/column), GR/DAuNSs/Cys/AuNPs/PD/GOx (3, pink line/column), GR/DAuNSs/Cys/PD/GOx (4, green line/column), and GR/Cys/PD/GOx (5, gray line/column). (B) Maximal power density calculated at 49.0 kΩ of an external resistance.

It was observed that the power densities of all bioanodes increased with increasing external load resistance until approximately 49 kΩ, and then started to decrease. The hybrid G-EBFCs based on the GR/Cys/AuNPs/PD/GOx bioanode (4.33 ± 0.55 μW cm^–2^) exhibited 1.17, 1,30, 1.51, and 1.83 times higher power density than that of the GR/AuNPs/Cys/PD/GOx (3.69 ± 0.29 μW cm^–2^), GR/DAuNSs/Cys/AuNPs/PD/GOx (3.33 ± 0.19 μW cm^–2^), GR/DAuNSs/Cys/PD/GOx (2.87 ± 0.24 μW cm^–2^), and GR/Cys/PD/GOx (2.36 ± 0.26 μW cm^–2^) bioanodes, respectively (Figure 3B). This indicates that gold nanocompounds enhance the electroactivity of the hybrid G-EBFCs, which corresponds to the information determined for other BFCs.^3,29^ The power densities registered by the here developed GR/DAuNSs/Cys/PD/GOx, GR/DAuNSs/Cys/AuNPs/PD/GOx, GR/AuNPs/Cys/PD/GOx, and GR/Cys/AuNPs/PD/GOx bioanodes were 19.9, 23.1, 25.6, and 30.1 times higher than that declared for the hybrid G-EBFCs based on a two-enzyme-based cascade (pyrroloquinoline quinone (PQQ)-dependent glucose dehydrogenase and 2-gluconate dehydrogenase) (0.144 ± 0.024 μW cm^–2^).^25^ The GR/Cys/AuNPs/PD/GOx, GR/AuNPs/Cys/PD/GOx, and GR/DAuNSs/Cys/AuNPs/PD/GOx bioanodes demonstrated good performance and were chosen for the design and subsequent studies.

### Evaluation of Electrocatalytic Activity and Performance of Selected Bioanodes

The performance of the electrochemical biosensors and bioelectrodes of enzymatic biofuel cells significantly depends on the structure and the orientation of the enzyme immobilized on the electrode surface, the location, and chemical nature of enzyme’s redox center.^3,13^ The *S*_EASA_ values of the GR/Cys/AuNPs/PD/GOx, GR/AuNPs/Cys/PD/GOx, and GR/DAuNSs/Cys/AuNPs/PD/GOx bioanodes were evaluated according to the methodology described in the Experimental Section. As shown in Figure S3, cyclic voltammograms were characterized by reversible redox peaks, which increased as the potential sweep rate increased. The *S*_EASA_ values of the GR/AuNPs/Cys/PD/GOx, GR/Cys/AuNPs/PD/GOx, and GR/DAuNSs/Cys/AuNPs/PD/GOx bioanodes were evaluated taking into the account the slope of the lines (Figure S4), and were determined to be 0.507, 0.538, and 0.589 cm^2^, respectively. The obtained *S*_EASA_ values were over 1.49 times higher than those for the PG/COOH-MWCNT/GOx (0.34 cm^2^).^22^

The impact of the potential sweep rates (from 0.150 to 0.010 V s^–1^) on the electrocatalytic activities of the GR/Cys/AuNPs/PD/GOx, GR/AuNPs/Cys/PD/GOx, and GR/DAuNSs/Cys/AuNPs/PD/GOx electrodes was evaluated using cyclic voltammograms (Figure S5) and is summarized in Figure 4A.

**Figure 4:**
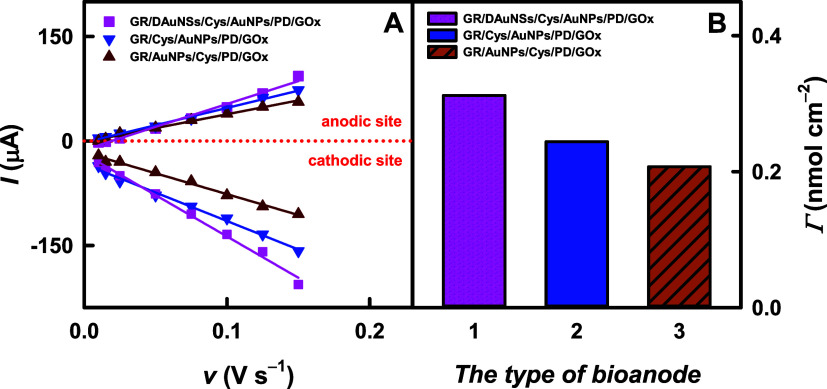
Influence of potential sweep rates on registered anodic and cathodic peak currents (A), and the effects of the GR/AuNPs/Cys/PD/GOx, GR/Cys/AuNPs/PD/GOx, GR/DAuNSs/Cys/AuNPs/PD/GOx bioanodes on the surface concentration of immobilized GOx (B). Current responses were registered in 0.05 mol L^–1^ SA buffer (pH 6.0) in the presence of 10 mmol L^–1^ glucose on the GR/DAuNSs/Cys/AuNPs/PD/GOx (1 dotted column, pink color), GR/Cys/AuNPs/PD/GOx (2 column, blue color), and GR/AuNPs/Cys/PD/GOx electrodes (3 shaded column, brown color).

As observed from the presented data, the redox peak currents of differently modified electrodes increase directly with the potential sweep rate of the electrode. This effect confirms the electrocatalytic efficiency and quasi-reversible redox behavior of the developed bioanodes.^17^ The surface concentration of the GOx biocomposites on GR modified with the electroactive species was calculated from the slopes of the ‘anodic lines’ (Figure 4A) using the Brown–Anson equation (eq 2). As shown in Figure 4B, the surface concentrations of GOx immobilized on the GR/AuNPs/Cys/PD/GOx, GR/Cys/AuNPs/PD/GOx, and GR/DAuNSs/Cys/AuNPs/PD/GOx electrodes were found to be 0.207, 0.244, and 0.312 nmol cm^–2^, respectively. The value of Γ determined for the GR/DAuNSs/Cys/AuNPs/PD/GOx electrode was 1.29 times higher than that of the PG/COOH-MWCNT/GOx electrode (0.242 nmol cm^–2^).^22^ This indicates that the combination of branched DAuNSs and AuNPs enables the achievement of a higher surface concentration of GOx.

Cyclic voltammograms registered by the GR/AuNPs/Cys/PD/GOx, GR/Cys/AuNPs/PD/GOx, and GR/DAuNSs/Cys/AuNPs/PD/GOx electrodes in 0.05 mol L^–1^ SA buffer (pH 6.0) in the absence and presence of 10 mmol L^–1^ glucose at 0.1 V s^–1^ were characterized by the reversibility of the redox reaction (Figure S6). As seen from the data, in the presence of 10 mmol L^–1^ glucose, the PD oxidation peak appeared at a potential of −0.040 mV vs. Ag/AgCl_(3 mol L^–1^_ _KCl)_ on GR/AuNPs/Cys/PD/GOx, −0.118 mV vs. Ag/AgCl_(3 mol L^–1^_ _KCl)_ on GR/Cys/AuNPs/PD/GOx, and −0.282 mV vs. Ag/AgCl_(3 mol L^–1^_ _KCl)_ on GR/DAuNSs/Cys/AuNPs/PD/GOx electrodes. The current responses of the anodic peaks for 10 mmol L^–1^ glucose registered by the GR/AuNPs/Cys/PD/GOx, GR/Cys/AuNPs/PD/GOx, and GR/DAuNSs/Cys/AuNPs/PD/GOx electrodes reached 39 μA (Figure S6A), 45.7 μA (Figure S6B), and 48.0 μA (Figure S6C), respectively. The difference between the anodic peaks registered in the absence and presence of 10 mmol L^–1^ glucose by the GR/DAuNSs/Cys/AuNPs/PD/GOx electrode was 24.4 μA, which was 1.21 times higher than that registered by the GR/AuNPs/Cys/PD/GOx (20.2 μA) electrode, and similar to that determined by the GR/Cys/AuNPs/PD/GOx electrode (24.1 μA). These results indicate that gold nanocompounds improve electron transfer efficiency.

The influence of glucose concentration on the current generated by the GR/Cys/AuNPs/PD/GOx, GR/AuNPs/Cys/PD/GOx, and GR/DAuNSs/Cys/AuNPs/PD/GOx bioanodes in 0.05 mol L^–1^ SA buffer (pH 6.0) at a 49.0 kΩ external load resistance was determined. The registered current slightly increased by increasing the concentration of glucose until 207 mmol L^–1^. The measured voltage and the applied 49.0 kΩ external load resistance were used in the calculation of the generated current by Ohm’s law. The calibration plots, the linear range, and diagrams of the current values for the developed hybrid G-EBFCs are presented in Figures 5A, B, and C, respectively. It is evident that the generated current increases with an increasing concentration of dissolved oxygen, which is involved in the catalytic action of GOx.^15^ As shown in Figures 5A,C, the presence of AuNPs advanced the electrochemical performance of the assessed bioanodes. The value of the current registered by the GR/DAuNSs/Cys/AuNPs/PD/GOx-based bioanode (−3.65 ± 0.36 μA) in the presence of 207 mmol L^–1^ glucose was 1.05 and 1.14 times lower than that determined for the GR/AuNPs/Cys/PD/GOx-based bioanode (−3.83 ± 0.09 μA) and GR/Cys/AuNPs/PD/GOx-based bioanode (−4.16 ± 0.22 μA), respectively. The slower charge transfer kinetics observed during the assessment of the GR/DAuNSs/Cys/AuNPs/PD/GOx-based bioanode might be attributed to the hindered electron transport caused by the presence of a dielectric Cys layer between the DAuNSs- and AuNPs-based structures.

**Figure 5:**
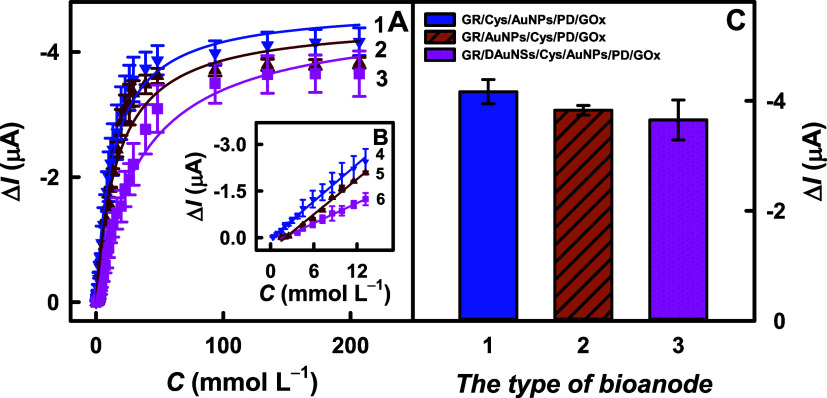
Anodic current dependencies (A), the linear range (B) of the GR/Cys/AuNPs/PD/GOx, GR/AuNPs/Cys/PD/GOx, and GR/DAuNSs/Cys/AuNPs/PD/GOx bioanodes at different glucose concentrations, and diagrams of the anodic current value of the assessed bioanodes in the presence of 207 mmol L^–1^ glucose (C). Details of the data: A, B, and C - the GR/Cys/AuNPs/PD/GOx bioanode (5A line 1, 5B line 4, and 5C column 1 (blue color)), the GR/AuNPs/Cys/PD/GOx (5A line 2, 5B line 5, and 5C shaded column 2 (brown color)), and the GR/DAuNSs/Cys/AuNPs/PD/GOx (5A line 3, 5B line 6, and 5C dotted column 3 (pink color)). All measurements were performed in 0.05 mol L^–1^ SA buffer (pH 6.0) using a 49.0 kΩ resistance, which was applied as an external load connected between the bioanode and cathode of the hybrid G-EBFCs.

The current densities, calculated taking into account the surface area of the assessed GR/DAuNSs/Cys/AuNPs/PD/GOx, GR/AuNPs/Cys/PD/GOx, and GR/Cys/AuNPs/PD/GOx bioanodes, were −51.4 ± 5.1, −53.9 ± 1.3, and −58.6 ± 3.1 μA cm^–2^, respectively. The current densities of the developed bioanodes were more than 1.63 times higher compared with the bioanode based on the two-enzyme cascade (PQQ-dependent glucose dehydrogenase and 2-gluconate dehydrogenase) (0.928 ± 0.106 μA cm^–2^) or the bioanode based on the six-enzyme cascade (PQQ-dependent glucose dehydrogenase, 2-gluconate dehydrogenase, aldolase, alcohol dehydrogenase, aldehyde dehydrogenase, and oxalate oxidase) (31.5 ± 6.5 μA cm^–2^) developed by Xu and Minteer.^25^ Demurtas et al. observed a current density of 37.7 μA cm^–2^ for the G-EBFC based on the bioanode prepared by drop-casting a Crassicarpon hotsonii cellobiose dehydrogenase (ChCDH) on NPG, polymerized with osmium polymer (Os­(bpy)_2_PVI) and methacryloyloxyethyl phosphorylcholine (MPC), cross-linked with poly­(ethylene glycol)­diglycidyl ether (PEDGE) - NPG/ChCDH/Os­(bpy)_2_PVI/PEDGE/MPC, and the biocathode prepared by the immobilization of a magnaporthe oryzae bilirubin oxidase (MoBOD) on a 3-mercaptopropionic acid (MPA) self-assembled monolayer with NPG and coated with N-ethyl-N′-(3-(dimethylamino)­propyl) carbodiimide hydrochloride (EDC) and *N*-hydroxysuccinimide (NHS) with MPC (NPG/MPA/MoBOD/EDC-NHS/MPC).^14^

A comparison of the hybrid G-EBFCs developed/assessed in this study, which were based on the GR/Cys/AuNPs/PD/GOx, GR/AuNPs/Cys/PD/GOx, and GR/DAuNSs/Cys/AuNPs/PD/GOx bioanodes, with enzymatic BFCs designed by other authors, is presented in Table 1.

**Table 1:** Comparison of the Performance of Some Enzymatic Glucose Biofuel Cells^a^,^b^,^c^

types of anodes and cathodes	power density (μW cm^–2^)	current density (μA cm^–2^)	stability (days) (retained response (%))	reference
Toray paper/two-enzyme cascade and Pt	0.144 ± 0.024	0.928 ± 0.106		^25^
Toray paper/six-enzyme cascade and Pt	6.74 ± 1.43	31.5 ± 6.5		
PG/COOH-MWCNTs/GOx and Pt	0.789	38.0 ± 0.7		^22^
NPG/ChCDH/Os(bpy)_2_PVI/PEDGE/MPC and NPG/MPA/MoBOD/EDC-NHS/MPC	4.4	37.7	0.042 (50)	^14^
GC/VC/γ-Fe_2_O_3_NP/GOx and C/PtNPs	30	0.165^c^	2 (50)	^21^
Au-*co*-Pt and BP/Au/MWCNTs/BOx	46.31	190.99	14 (30)	^23^
Au/MWCNTs/Pd_plate_/GOx-PABA-PdNPs/CS and Au/MWCNTs/Pt	54.5	1.25^c^	30 (78.9)	^20^
GC/graphene-CS-GOx-Fc-MeOH and Pt	69.1	4.21^c^	7 (62)	^24^
VC cloth/AuNPs-PtNPs and VC paper/Pt	58.2^c^	150^c^	0.125 (72.6)	^29^
GC/PANI-Ag/Frt/GOx and Pt		25.4 ± 2.0^c^	10 (88)	^17^
GR/DAuNSs/Cys/AuNPs/PD/GOx and Pt	3.33 ± 0.19	–51.4 ± 5.1	5 (50)	This work
GR/AuNPs/Cys/PD/GOx and Pt	3.69 ± 0.29	–53.9 ± 1.3	25 (50)	This work
GR/Cys/AuNPs/PD/GOx and Pt	4.33 ± 0.55	–58.6 ± 3.1	9 (50)	This work

a Au, gold; Au-*co*-Pt, gold wire modified with colloidal platinum; AuNPs, gold nanoparticles; BOx, bilirubin oxidase; BP, Buckypaper; ChCDH, Crassicarpon hotsonii cellobiose dehydrogenase; CS, chitosan; COOH-MWCNTs, carboxylic acid-functionalized multiwalled carbon nanotubes; Cys, cystamine; γ-Fe_2_O_3_NP, maghemite nanoparticle; EDC-NHS, N-ethyl-N′-(3-(dimethylamino)­propyl) carbodiimide hydrochloride and *N*-hydroxysuccinimide; DAuNSs, dendritic gold nanostructures; Fc-MeOH, ferrocene methanol; Frt, ferritin; GC, glassy carbon; GOx, glucose oxidase; GOx-PABA-PdNPs, a biocomposite of glucose oxidase, poly­(3-anilineboronic acid), and palladium nanoparticles; GR, graphite rod; MoBOD, magnaporthe oryzae bilirubin oxidase; MPA, 3-mercaptopropionic acid; MPC, methacryloyloxyethyl phosphorylcholine; MWCNTs, multiwalled carbon nanotubes; NPG, nanoporous gold; Os­(bpy)_2_PVI, osmium polymer; PANI-Ag, nanocomposites of polyaniline and silver; PD, 1,10-phenathroline-5,6-dione; Pd_plate_, electroplated palladium; PEDGE, poly­(ethylene glycol) diglycidyl ether; PG, pencil graphite; Pt, platinum; PtNPs, platinum nanoparticles; six-enzyme-based cascade, pyrroloquinoline quinone (PQQ)-dependent glucose dehydrogenase, 2-gluconate dehydrogenase, aldolase, alcohol dehydrogenase, aldehyde dehydrogenase, and oxalate oxidase; two- enzyme-based cascade, PQQ-dependent glucose dehydrogenase and 2-gluconate dehydrogenase; VC, ''Vulcan'' carbon.

b The dimension is expressed in mA cm^–2^.

c The dimension is expressed in mW cm^–2^.

The analytical performance of bioanodes, including the linear range, sensitivity, limit of detection, and stability, is important if such electrodes are simultaneously used in the development of biosensing systems. As shown in Figure 5B, hybrid G-EBFCs based on differently modified bioanodes exhibited a broad linear range (up to 13.0 mmol L^–1^) with a high determination coefficient (not less than *R* ^2^ = 0.9913). The developed G-EBFCs were simultaneously suitable for biosensing and were similar to those of G-EBFCs based on the Au/MWCNTs/Pd_plate_/GOx-PABA-PdNPs/CS bioanode and Au/MWCNTs/Pt cathode (up to 13.5 mmol L^–1^).^20^

A hybrid glucose biofuel cell based on gold wire electrochemically modified with colloidal platinum (Au-*co*-Pt) as anode, and Buckypaper (BP) coated with gold, MWCNTs, bilirubin oxidase (BP/Au/MWCNTs/BOx) as cathode was characterized by a linear range up to 20 mmol L^–1^ with a low *R* ^2^ of 0.9653.^23^ The broad linear range G-EBFCs reported in the present research demonstrates the suitability of the G-EBFCs for practical applications. The GR/AuNPs/Cys/PD/GOx, GR/Cys/AuNPs/PD/GOx, and GR/DAuNSs/Cys/AuNPs/PD/GOx bioanodes were characterized by 4.78, 7.00, and 11.3% reproducibility for the determination of 38.4 mmol L^–1^ glucose.

The developed hybrid G-EBFC based on the GR/Cys/AuNPs/PD/GOx bioanode, with a sensitivity of 2.80 μA mM^–1^ cm^–2^, was 1.05 and 1.84 times more sensitive than G-EBFCs based on the GR/AuNPs/Cys/PD/GOx bioanode with a sensitivity of 2.66 μA mM^–1^ cm^–2^ and on the GR/DAuNSs/Cys/AuNPs/PD/GOx bioanode with a sensitivity of 1.52 μA mM^–1^ cm^–2^. The low sensitivity of the GR/DAuNSs/Cys/AuNPs/PD/GOx bioanode can be explained by the formation of an insulating Cys layer between the DAuNSs and AuNPs layers, which reduces the electron transfer efficiency due to the formation of an additional insulating barrier.

The limits of detection for the GR/Cys/AuNPs/PD/GOx bioanode (0.021 mmol L^–1^) and for the GR/AuNPs/Cys/PD/GOx bioanode (0.022 mmol L^–1^) were comparable and were, respectively, 3.86 and 3.68 times lower than that determined for the GR/DAuNSs/Cys/AuNPs/PD/GOx bioanode (0.081 mmol L^–1^). This indicates the advantage of G-EBFCs modified with AuNPs compared to those incorporating a combination of DAuNSs/Cys/AuNPs layered structures.

The long lifetime of BFCs is considered one of the main challenges.^4^ The storage stability of hybrid G-EBFCs based on the GR/DAuNSs/Cys/AuNPs/PD/GOx, GR/Cys/AuNPs/PD/GOx, and GR/AuNPs/Cys/PD/GOx bioanodes was evaluated after storing in a refrigerator, over the SA buffer (pH 6.0), between measurements for 21, 38, and 54 days, respectively. The variation in the current over time using differently modified bioanodes is presented in Figure 6A. It is evident that the GR/DAuNSs/Cys/AuNPs/PD/GOx, GR/Cys/AuNPs/PD/GOx, and GR/AuNPs/Cys/PD/GOx bioanodes retained 50% of their initial responses after 5, 9, and 25 days, respectively. The GR/AuNPs/Cys/PD/GOx bioanode is 5.0 and 2.78 times more stable than the GR/DAuNSs/Cys/AuNPs/PD/GOx and GR/Cys/AuNPs/PD/GOx bioanodes, respectively. The lower storage stability of the GR/DAuNSs/Cys/AuNPs/PD/GOx bioanode may be explained by the formation of multiple layers and the possible partial detachment of the mechanically fragile DAuNS-containing layer from the GR surface. This explanation may be considered hypothetical because the morphology of the GR/DAuNSs/Cys/AuNPs/PD/GOx bioanode was not determined after the stability experiments. For the GR/Cys/AuNPs/PD/GOx bioanode, the attachment of Cys to the graphite rod is typically weaker than to the Au–S bonds, which may result in less stable immobilization of AuNPs. In contrast, the higher storage stability of the GR/AuNPs/Cys/PD/GOx bioanode can be attributed to the binding of Cys to AuNPs via thiol groups by the self-assembled monolayer of Cys with AuNPs formation,^33^ creating a more favorable environment for GOx immobilization.

**Figure 6:**
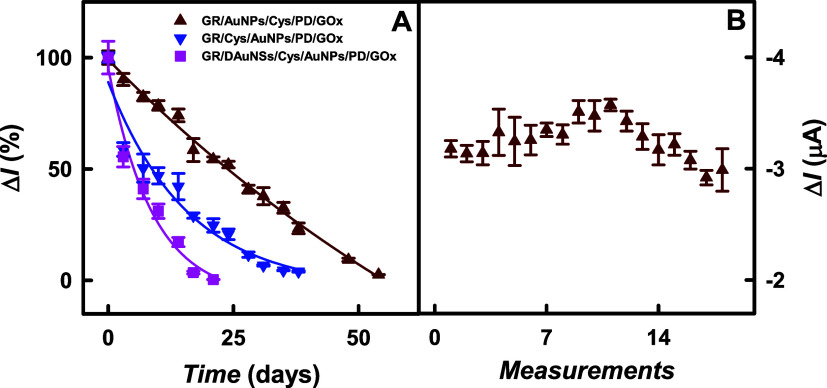
Current variation over time for differently modified bioanodes (A) and the repeatability of the GR/AuNPs/Cys/PD/GOx bioanode during 18 repeated measurements (B). The measurements presented in graph A were performed in the presence of 93.5 mmol L^–1^ glucose using GR/AuNPs/Cys/PD/GOx (brown line), GR/Cys/AuNPs/PD/GOx (blue line), and GR/DAuNSs/Cys/AuNPs/PD/GOx (pink line). The measurements shown in graph B were performed in the presence of 50.0 mmol L^–1^ glucose. All measurements were performed in a 0.05 mol L^–1^ SA buffer (pH 6.0) using a 49.0 kΩ external resistance.

The G-EBFC based on the GR/AuNPs/Cys/PD/GOx bioanode was 12.5 times more stable than the G-EBFC consisting of a bioanode based on a mixture of MWCNTs, GOx, and naphthoquinone, and a biocathode based on a mixture of horseradish peroxidase and MWCNTs^31^ or than the G-EBFC based on the GC/VC/γ-Fe_2_O_3_NP/GOx bioanode and on the C/PtNPs cathode,^21^ which were active only within 2 days. The 50% of initial power density registered using the EBFC based on the NPG/ChCDH/Os­(bpy)_2_PVI/PEDGE/MPC as a bioanode and the NPG/MPA/MoBOD/EDC-NHS/MPC as a biocathode decayed after 1 h (0.042 days).^14^ The G-EBFCs based on Au-*co*-Pt as an anode and BP/Au/MWCNTs/BODx as a biocathode retained about 30% of their initial power density after 14 days,^23^ and the G-EBFCs based on the GC/graphene-CS-GOx-Fc-MeOH as a bioanode and Pt as a cathode of 62% after 7 days.^24^ The GR/AuNPs/Cys/PD/GOx bioanode was characterized by a good repeatability of 5.37% for 18 measurements (Figure 6B) and a 3 s measurement duration.

The hybrid G-EBFC based on the GR/AuNPs/Cys/PD/GOx bioanode, characterized by a low limit of detection, good sensitivity, and storage stability, is determined to be a more suitable biosensing system for glucose determination in real samples. This G-EBFC can be used for glucose monitoring in medical, food, and beverage samples.

### Application of Selected Bioanodes for the Determination of Glucose in Real Samples

Interfering compounds may undergo oxidation during electrochemical glucose biosensing, leading to inaccurate analyte detection.^45–48^ Therefore, the influence of interfering compounds on the selectivity of the hybrid G-EBFC based on the GR/AuNPs/Cys/PD/GOx bioanode was performed according to the methodology described in the Experimental Section and in the Supporting Information. Figure S7A demonstrates that the GR/AuNPs/Cys/PD/GOx bioanode retains its performance only toward glucose in the presence of 1.0 mmol L^–1^ of other saccharides (fructose, mannose, saccharose, galactose, and xylose) dissolved in human serum. The effective anti-interference capability of the developed GR/AuNPs/Cys/PD/GOx bioanode was determined in several other real samples, as reported in Table 2.

**Table 2:** Currents Registered with the Hybrid G-EBFC Based on the GR/AuNPs/Cys/PD/GOx Bioanode in Real Samples

	current^a^ (%)					
	AA (mmol L^–1^)			UA (mmol L^–1^)		saccharides (mmol L^–1^)
real samples	0.01	0.05	0.1	0.01	0.05	1.0
Human serum	103	105	106	99.0	94.6	98.3
Human saliva	101	101	102	99.6	97.8	103
Wine	101	102	104			102
Coconut milk	102	103	104			103
Almond milk	101	102	103			101
Apple juice	101	102	102			101
Mandarin juice	100	101	101			101

a Currents were calculated using Ohm’s law after the voltage registration in 10-fold diluted with 0.05 mol L^–1^ SA buffer (pH 6.0) of human serum, and saliva, or 100-fold diluted wine, coconut and almond milk, as well as apple and mandarin juices using a 49.0 kΩ external resistance.

The physiological blood^49^ and saliva^50^ glucose concentrations of nondiabetic individuals are typically below 6.0 and 0.03 mmol L^–1^, whereas in diabetic patients’ blood^6^ and saliva^50^ it can reach up to 30 and 0.21 mmol L^–1^, respectively. Blood glucose concentrations substantially exceed the possible concentrations of ascorbic^51^ and uric^52^ acids (–0.141 and 0.1 mmol L^–1^, respectively). The influence of electroactive species on the current of the hybrid G-EBFC based on the GR/AuNPs/Cys/PD/GOx bioanode in human serum is presented in Figure S7B and Table 2. The calculated currents generated in the presence of AA and UA are expressed as a percentage of the glucose current, which was estimated to be 100%. It can be observed that the current increased after the addition of 10 mmol L^–1^ glucose with 0.1 mmol L^–1^ ascorbic acid (the investigated AA concentration is 7.09 times higher than that in 10-fold diluted human serum), which was smaller than 6.0% of the relative value determined in the absence of AA. No significant influence (less than 4.0%) on the current of the GR/AuNPs/Cys/PD/GOx bioanode was observed during the assessment of all other real samples (Table 2).

Uric acid exhibited a negligible effect on the performance of the hybrid G-EBFC based on the GR/AuNPs/Cys/PD/GOx bioanode. After the addition of a solution containing 10 mmol L^–1^ glucose and 0.01 or 0.05 mmol L^–1^ UA in human serum, the current decreased by 1.0 or 5.4%, respectively, relative to that obtained without UA. The impact of 0.05 mmol L^–1^ UA (5 times higher concentration than that present in 10-fold diluted human serum). A lower effect of UA influence was observed in human saliva, where the currents decreased only by 0.40 or 2.2% after the addition of a solution containing 10 mmol L^–1^ glucose with 0.01 or 0.05 mmol L^–1^ UA, respectively. The good anti-interference ability of the GR/AuNPs/Cys/PD/GOx bioanode indicates its suitability for glucose biosensing applications in real samples.

The developed G-EBFC based on the GR/AuNPs/Cys/PD/GOx bioanode was applied for glucose detection in diluted real samples using the ‘standard addition’ method. The measurements were carried out in 10-fold diluted human serum containing 0.484 mmol L^–1^ glucose, which was detected using a commercial glucometer (Contour plus, Bayer Consumer Care AG, Basel, Switzerland). The detected glucose concentration was 0.458 ± 0.029 mmol L^–1^ with a recovery ratio of 94.6 ± 6.0% (Figure S8). Good compatibility between the developed hybrid G-EBFC based on the GR/AuNPs/Cys/PD/GOx bioanode and commercial sensor signals is observed. The determination of glucose in real samples is presented in Table 3.

**Table 3:** Recovery of Glucose Determination in Real Samples by the GR/AuNPs/Cys/PD/GOx Bioanode

	glucose concentration (mmol L^–1^)		
real samples	added	detected^a^	recovery ratio (%)
Human serum	2.48	2.35 ± 0.14	94.8
	5.48	5.24 ± 0.15	95.6
	8.48	8.27 ± 0.30	97.5
Human saliva	2.00	1.91 ± 0.07	95.5
	3.00	2.86 ± 0.09	95.3
	4.50	4.41 ± 0.30	98.0
Wine	2.30	2.24 ± 0.16	97.4
	7.20	7.07 ± 0.10	98.2
Coconut milk	2.00	1.94 ± 0.10	97.0
	4.50	4.41 ± 0.25	98.0
Almond milk	2.00	1.87 ± 0.18	93.5
	3.00	2.80 ± 0.18	93.3
Apple juice	2.00	1.93 ± 0.06	96.5
	3.00	2.91 ± 0.04	97.0
Mandarin juice	2.00	1.93 ± 0.04	96.5
	3.00	2.92 ± 0.14	97.3

a All measurements were performed after 10-fold dilution with 0.05 mol L^–1^ SA buffer (pH 6.0) of human serum, and saliva, or 100-fold dilution of wine, coconut and almond milk, as well as apple and mandarin juices using a 49.0 kΩ external resistance.

The recovery ratio for hybrid G-EBFC based on the GR/AuNPs/Cys/PD/GOx bioanode was in the following ranges: (i) for human serum, from 94.8 ± 5.7 to 97.5 ± 3.5%; (ii) for human saliva, from 95.3 ± 3.0 to 98.0 ± 6.7%, (iii) for wine, from 97.4 ± 7.0 to 98.2 ± 1.4%; (iv) for coconut milk, from 97.0 ± 5.0 to 98.0 ± 5.6%; (v) for almond milk, from 93.3 ± 6.0 to 93.5 ± 9.0%; (vi) for apple juice, from 96.5 ± 3.0 to 97.0 ± 1.3%; and (vii) for mandarin juice, from 96.5 ± 2.0 to 97.3 ± 4.7%, which indicates their suitability for application in real sample analysis.

The developed hybrid G-EBFC based on the GR/AuNPs/Cys/PD/GOx bioanode exhibits an analytical performance comparable to that of commercial sensors dedicated to glucose determination in human serum. Glucose concentration determination standards established by some countries and regulatory agencies are slightly different: (i) in the United States of America, the accuracy criterion in ISO 15197:2013 is 98 ± 15% for ≥75 mg dL^–1^ (≥4.17 mmol L^–1^) of glucose; (ii) in Europe, it is 95 ± 15% for ≥100 mg dL^–1^ (≥5.55 mmol L^–1^), and (iii) in Australia, it is 99% for diabetic patients (Type 1 diabetes).^53^

In this research, the developed membraneless hybrid G-EBFC based on the GR/AuNPs/Cys/PD/GOx bioanode demonstrates economic viability, resulting from the minimal chemical consumption during fabrication and the employment of straightforward, improved, cost-efficient equipment: a potentiostat is not needed, and only two electrodes instead of three are used. The developed G-EBFC is advantageous because of (i) sufficient power density (3.69 ± 0.29 μW cm^–2^), which is determined by the relatively high surface concentration of immobilized GOx (0.207 nmol cm^–2^); (ii) long-term stability (50% of initial current persists for a period of up to 25 days); (iii) additional applicability of G-EBFC in glucose-sensor mode with rather good sensitivity (2.66 μA mM^–1^ cm^–2^), reproducibility (4.78%), and repeatability (5.37%), low limit of detection (0.022 mmol L^–1^), sufficient linear range (up to 13.0 mmol L^–1^), short duration of measurement (of 3 s), and good anti-interference capability. All these advantages pave the way for the application of the developed G-EBFC in powering biomedical devices and/or for biomedical diagnostics and monitoring of beverage quality. G-EBFC-based systems may be useful for both *in vitro* and *in vivo* applications.

## Conclusions

During the last few decades, glucose biosensing has gained widespread attention due to the increasing prevalence of diabetes and recent advancements in biosensor technology. This study highlights the development of a relatively inexpensive and nontoxic membraneless glucose enzymatic biofuel cell based on graphite rod electrodes modified with DAuNSs and/or AuNPs and a Cys-based self-assembled monolayer. The membraneless hybrid G-EBFCs based on the GR/Cys/AuNPs/PD/GOx, GR/AuNPs/Cys/PD/GOx, and GR/DAuNSs/Cys/AuNPs/PD/GOx bioanodes were the most efficient. The power density of the developed G-EBFCs was not very high but was comparable to that of other reported carbon-based systems. Although the two hybrid G-EBFCs, which were based on the GR/Cys/AuNPs/PD/GOx and GR/AuNPs/Cys/PD/GOx bioanodes, exhibit similar sensitivity and a low limit of detection, the application of the GR/AuNPs/Cys/PD/GOx bioanode provides significantly enhanced storage stability of G-EBFC. The good anti-interference capability of the hybrid G-EBFC based on the GR/AuNPs/Cys/PD/GOx bioanode was applied for the determination of glucose in real samples, such as human serum, saliva, and some beverages. The methodology presented in this study demonstrates the potential advantages of G-EBFC. The developed G-EBFC represents a promising platform for future research in the area of self-powered biosensing systems for glucose determination in the fluids of diabetic patients.

## Supplementary Material



## Notes

Colloidal solution and the absorbance spectrum of 13 nm AuNPs (Figure S1); FE-SEM images of electrochemically synthesized DAuNSs on the GR surface (Figure S2); cyclic voltammograms of the GR/AuNPs/Cys/PD/GOx, GR/Cys/AuNPs/PD/GOx, and GR/DAuNSs/Cys/AuNPs/PD/GOx electrodes recorded at potential sweep rates ranging from 0.175 to 0.050 V s^–1^ (Figure S3); relationship between the square root of the potential sweep rate and peak anodic current for the GR/AuNPs/Cys/PD/GOx, GR/Cys/AuNPs/PD/GOx, and GR/DAuNSs/Cys/AuNPs/PD/GOx electrodes (Figure S4); cyclic voltammograms of the GR/AuNPs/Cys/PD/GOx, GR/Cys/AuNPs/PD/GOx, and GR/DAuNSs/Cys/AuNPs/PD/GOx electrodes recorded by applied potential sweep rate from 0.150 to 0.010 V s^–1^ (Figure S5); cyclic voltammograms of the GR/AuNPs/Cys/PD/GOx, GR/Cys/AuNPs/PD/GOx, and GR/DAuNSs/Cys/AuNPs/PD/GOx electrodes recorded in the absence and presence of glucose (Figure S6); influence of saccharides and electroactive species on the current of the hybrid G-EBFC based on the GR/AuNPs/Cys/PD/GOx bioanode (Figure S7); detection of glucose in a 10-fold diluted sample of human serum containing 0.484 mmol L^–1^ of glucose by the ‘standard addition’ method (Figure S8) (PDF)

## Abbrevations

A: surface area of the working electrode
AA: l-ascorbic acid
Au: gold
Au-*co*-Pt: gold wire modified with colloidal platinum
Au–S: gold–thiol bonds
α-Al_2_O_3_: α-aluminum oxide
AuNPs: gold nanoparticles
BFCs: biofuel cells
BODx: bilirubin oxidase
BP: Buckypaper
C: concentration of the electroactive species
ChCDH: Crassicarpon hotsonii cellobiose dehydrogenase
COOH-MWCNTs: carboxylic acid-functionalized multiwalled carbon nanotubes
CS: chitosan
CV: cyclic voltammetry
Cys: cystamine
γ-Fe_2_O_3_NP: maghemite nanoparticle
D: diffusion coefficient
DAuNSs: dendritic gold nanostructures
*E*: voltage
EDC: N-ethyl-N′-(3-(dimethylamino)­propyl) carbodiimide hydrochloride
*F*: Faraday constant
Fc-MeOH: ferrocene methanol
Frt: ferritin
GA: glutaraldehyde
GC: glassy carbon
G-EBFCs: glucose enzymatic biofuel cells
GOx: glucose oxidase
GR: graphite rod
Γ: surface concentration of immobilized GOx;
*I*: current
*I*_p_: maximum peak current
*J*: power density
LR: linear range
LOD: limit of detection
MoBOD: magnaporthe oryzae bilirubin oxidase
MPA: 3-mercaptopropionic acid
MPC: methacryloyloxyethyl phosphorylcholine
MWCNTs: multiwalled carbon nanotubes
*n*: number of electrons
NHS: *N*-hydroxysuccinimide
NPG: nanoporous gold
Os­(bpy)_2_PVI: osmium polymer
O_2_: oxygen
*P*: power output
PABA: poly­(3-anilineboronic acid)
PANI-Ag: nanocomposites of polyaniline and silver
PD: 1,10-phenathroline-5,6-dione
Pd_plate_: electroplated palladium
PEDGE: poly­(ethylene glycol) diglycidyl ether
PG: pencil graphite
Pt: platinum
PdNPs: palladium nanoparticles
PtNPs: platinum nanoparticles
PQQ: pyrroloquinoline quinone
*R*: resistance
*R*: ideal gas constant
*R* ^2^: correlation coefficient
Ru­(NH_3_)_6_Cl_3_: hexaammineruthenium­(III) chloride
SA: sodium acetate
SAM: self-assembled monolayer
*S*_EASA_: electroactive surface area of modified bioanodes
six-enzyme-based cascade: PQQ-dependent glucose dehydrogenase, 2-gluconate dehydrogenase, aldolase, alcohol dehydrogenase, aldehyde dehydrogenase, and oxalate oxidase
two enzyme-based cascade: PQQ-dependent glucose dehydrogenase and 2-gluconate dehydrogenase
*T*: temperature
UA: uric acid
*v*: potential sweep rate
VC: ''Vulcan'' carbon
