# Supplementary material for: Design and Characterization of Hybrid Glucose-Powered Enzymatic Biofuel Cells Based on the Combination of Different Gold-Based Nanocompounds
Source: Langmuir. 2026 Jul 4;42(28):20590–601. doi: 10.1021/acs.langmuir.6c02417 (PMC13394398; doi:10.1021/acs.langmuir.6c02417)
Supplement: Supplementary file 1 [file la6c02417_si_001.pdf]

Supporting Information
Design and Characterization of Hybrid Glucose-Powered Enzymatic Biofuel Cells Based on the Combination of Different Gold-Based Nanocompounds

Natalija German [1], Almira Ramanaviciene [1,2], Arunas Ramanavicius [2,*]

[1] Department of Immunology and Bioelectrochemistry, State Research Institute Centre for Innovative Medicine, Santariskiu 5, LT-08406, Vilnius, Lithuania

[2] NanoTechnas – Center of Nanotechnology and Materials Science, Faculty of Chemistry and Geosciences, Vilnius University, LT-03225, Vilnius, Lithuania

[*] *Corresponding author*: arunas.ramanavicius@chf.vu.lt

The Synthesis of 13 nm Gold Nanoparticles (AuNPs) and Dendritic Gold Nanostructures (DAuNSs)

To synthesize 13 nm AuNPs (50 μg mL^{-1}), placed in different Erlenmeyer flasks ((A): 0.0125% $HAuCl_4$ solution and (B) a solution containing 0.2 % trisodium citrate dihydrate, 0.00125 % tannic acid) were heated to +60°C with continuous magnetic stirring. After that, both solutions were mixed, heated until it boils using a magnetic stirring, and left to boil for a few minutes to yield a red-coloured solution (Figure S1A). Subsequently, the Erlenmeyer flask with colloidal solution of AuNPs (pH 6.5, an absorbance peak at 521 nm of a wavelength (Figure S1B)) was stored into the ice to complete the reduction process. The solution of 13 nm AuNPs was stored at +4°C prior to further measurements.

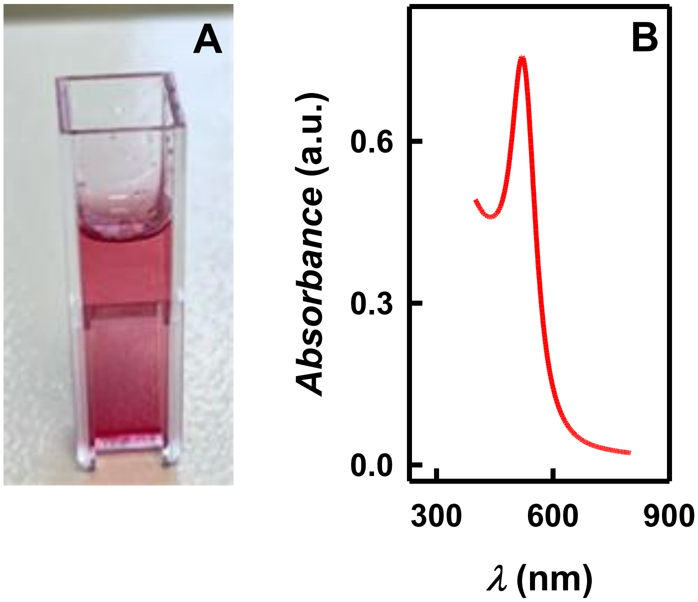

Figures S1. Colloidal solution (A) and the absorbance spectrum (B) of 13 nm AuNPs.

Electrochemical synthesis of DAuNSs was performed using a computerized potentiostat/galvanostat Autolab/PGSTAT 302N (EcoChemie, The Netherlands) with GPES 4.9 software (AUT83239) and a three-electrode system: the graphite rod (GR) as the working electrode, 2 cm^2 Pt spiral BASi Research Products (West Lafayette, IN, USA) as an auxiliary electrode, and $Ag/AgCl_{(3\ mol\ L^{-1}\ KCl)}$ from Metrhom (Herisau, Switzerland) as a reference electrode. Electrochemical synthesis of DAuNSs on the surface of GR was performed by constant potential amperometry method from constant stirring (1200 rpm) 6.0 mmol L^{-1} $HAuCl_4$ solution with 0.1 mol L^{-1} KNO_3 at –0.4 V of potential vs. $Ag/AgCl_{(3\ mol\ L^{-1}\ KCl)}$ for 400 s. The morphology and size of electrochemically synthesized DAuNSs on the GR surface were estimated by a high-resolution field emission scanning electron microscope Hitachi SU-70 (FE-SEM, Tokyo, Japan). As is seen from Figure S2, DAuNSs were electrochemically synthesized on the surface of the GR as long, thin, and branched aggregates.

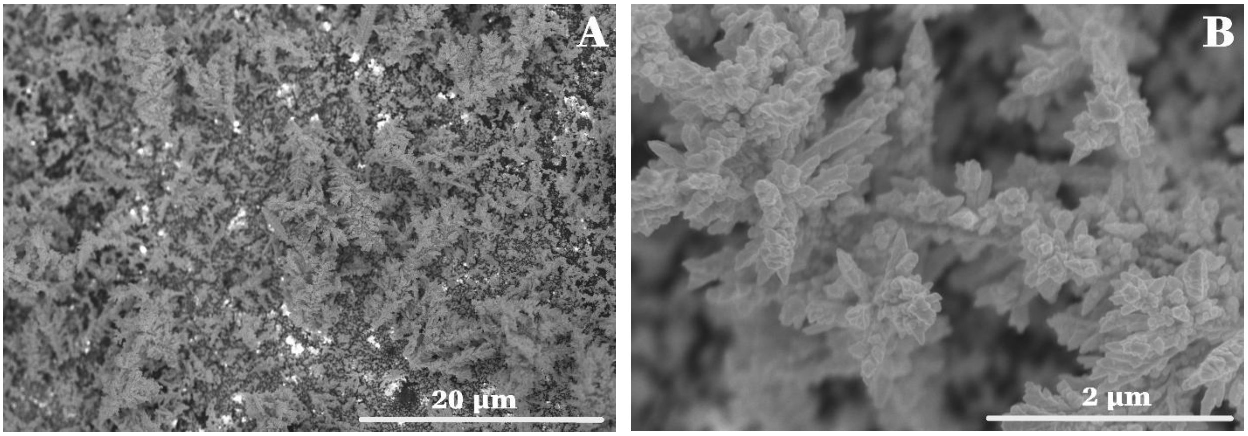

Figure S2. FE-SEM images of electrochemically synthesized DAuNSs on the GR surface. A, B – 20 and 2 µm of a scale, respectively; FE-SEM images were acquired at an accelerating voltage of 10 kV and a magnification of 25 k.

The Study of the Selectivity of Hybrid Glucose Enzymatic Biofuel Cell (G-EBFC) to Interfering Compounds

The practical uses of G-EBFC based on the GR/AuNPs/Cys/PD/GOx bioanode were performed in diluted by SA buffer (pH 6.0) real samples (human serum and saliva, wine, coconut and almond milk, apple and mandarin juices) and centrifuged by the IEC CL31R Multispeed centrifuge from Thermo Industries SAS (Aze Bellitourne, Château-Contier, France) during 8 min (14600 × g). The influence of saccharides on the current toward glucose was investigated in real samples with 10 and 20 mmol L^{-1} of glucose before and after the addition of 1.0 mmol L^{-1} of fructose, mannose, saccharose, galactose, and xylose. The impact of ascorbic acid (AA) and uric acid (UA) on the current toward glucose was measured in the samples of human serum and saliva, containing 10 mmol L^{-1} of glucose; both in the absence and presence of 0.01, 0.05, or 0.1 mmol L^{-1} AA, and 0.01, or 0.05 mmol L^{-1} UA.

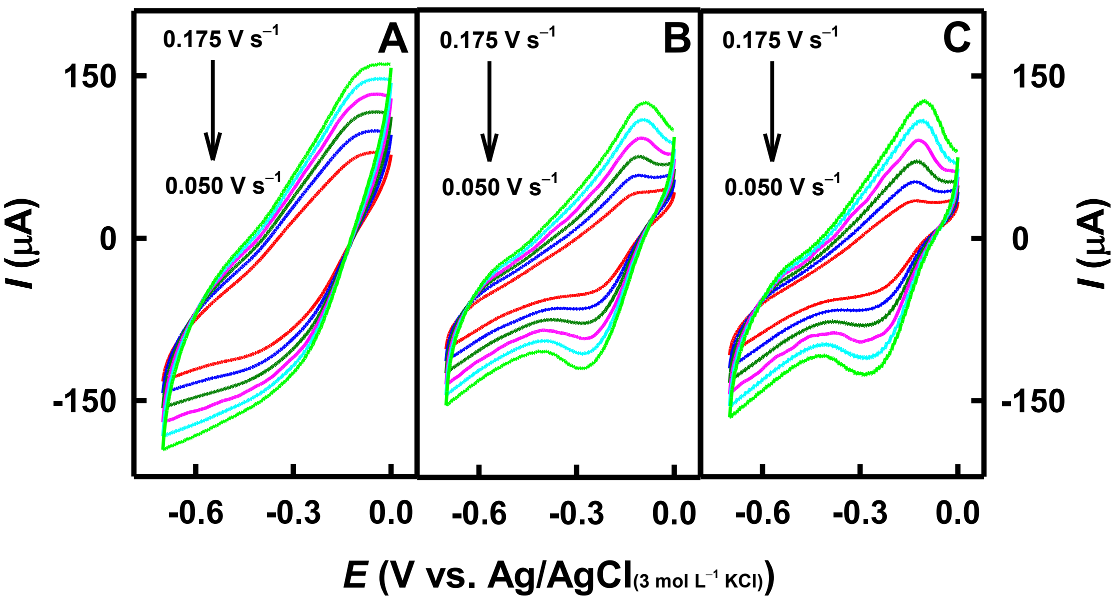

Figures S3. Cyclic voltammograms of the GR/AuNPs/Cys/PD/GOx (A), GR/Cys/AuNPs/PD/GOx (B), and GR/DAuNSs/Cys/AuNPs/PD/GOx (C) electrodes recorded at potential sweep rates ranging from 0.175 to 0.050 V s^{-1} (from bright green to red colours). The measurements were performed in 1.0 mmol L^{-1} $Ru(NH_3)_6Cl_3$ solution containing 0.1 mol L^{-1} KCl.

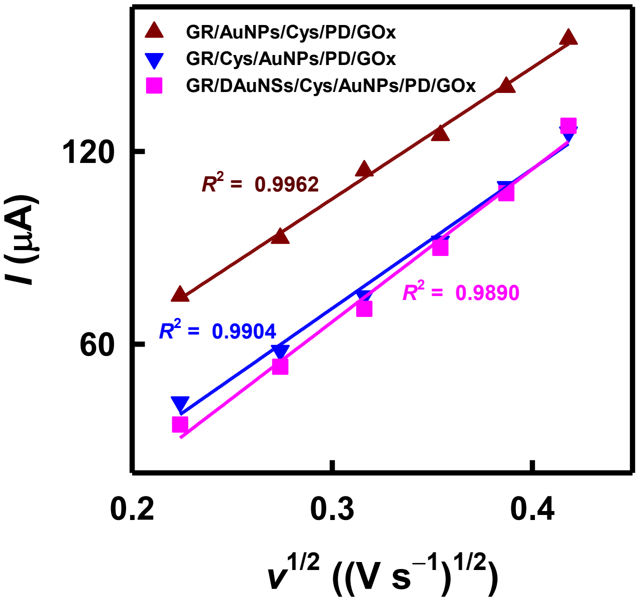

Figure S4. Relationship between the square root of the potential sweep rate and peak anodic current for the GR/AuNPs/Cys/PD/GOx (brown line), GR/Cys/AuNPs/PD/GOx (blue line), and GR/DAuNSs/Cys/AuNPs/PD/GOx (pink line) electrodes. The measurements were performed in 1.0 mmol L^{-1} $Ru(NH_3)_6Cl_3$ solution containing 0.1 mol L^{-1} KCl by CV.

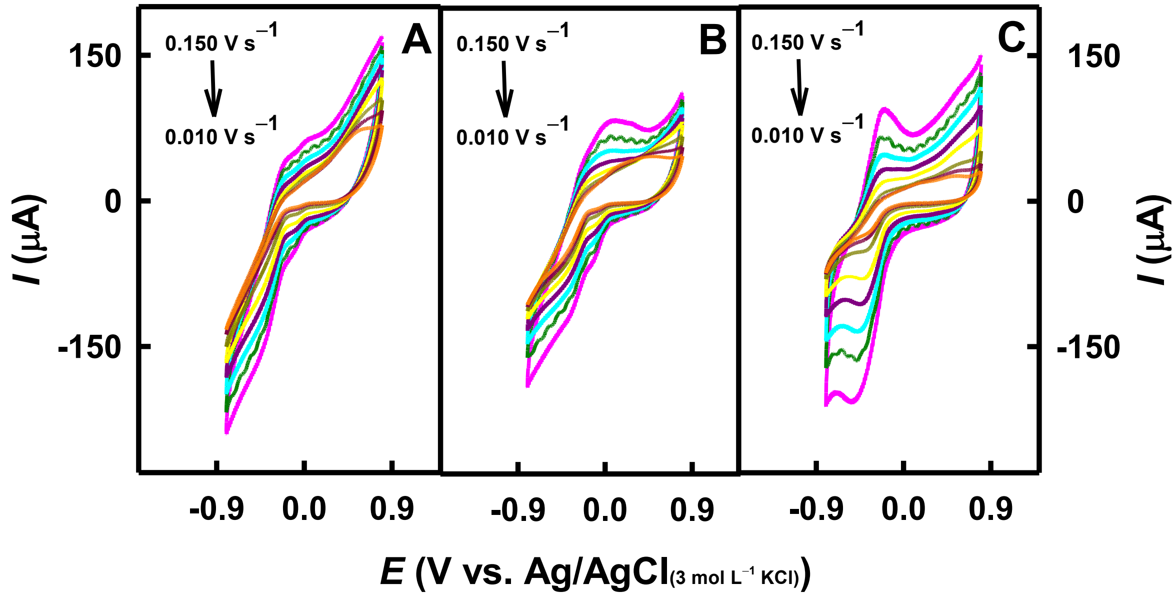

Figure S5. Cyclic voltammograms of the GR/AuNPs/Cys/PD/GOx (A), GR/Cys/AuNPs/PD/GOx (B), and GR/DAuNSs/Cys/AuNPs/PD/GOx (C) electrodes recorded by applied potential sweep rate from 0.150 to 0.010 V s^{-1} (from pink to orange colours). Cyclic voltammograms were registered in 0.05 mol L^{-1} SA buffer (pH 6.0) in the presence of 10 mmol L^{-1} glucose.

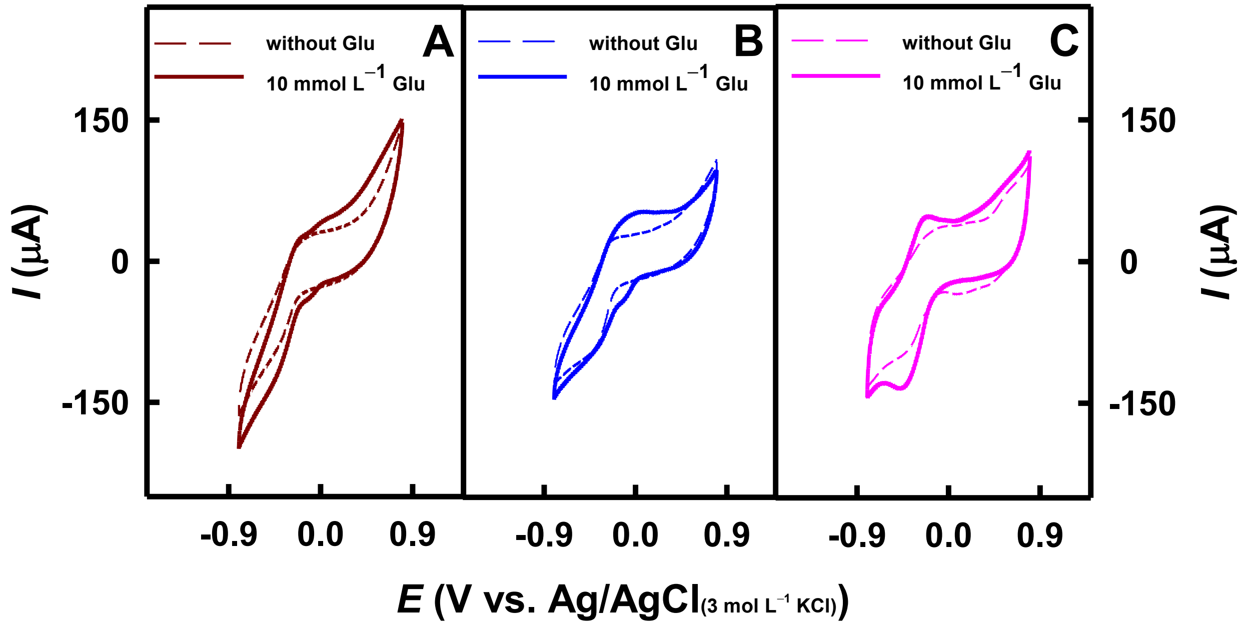

Figure S6. Cyclic voltammograms of the GR/AuNPs/Cys/PD/GOx (A), GR/Cys/AuNPs/PD/GOx (B), and GR/DAuNSs/Cys/AuNPs/PD/GOx (C) electrodes recorded in the absence and presence of glucose. Cyclic voltammograms were registered in 0.05 mol L^{-1} SA buffer (pH 6.0) in the absence (thin, dotted lines) and presence of 10 mmol L^{-1} glucose (solid lines) by 0.1 V s^{-1}.

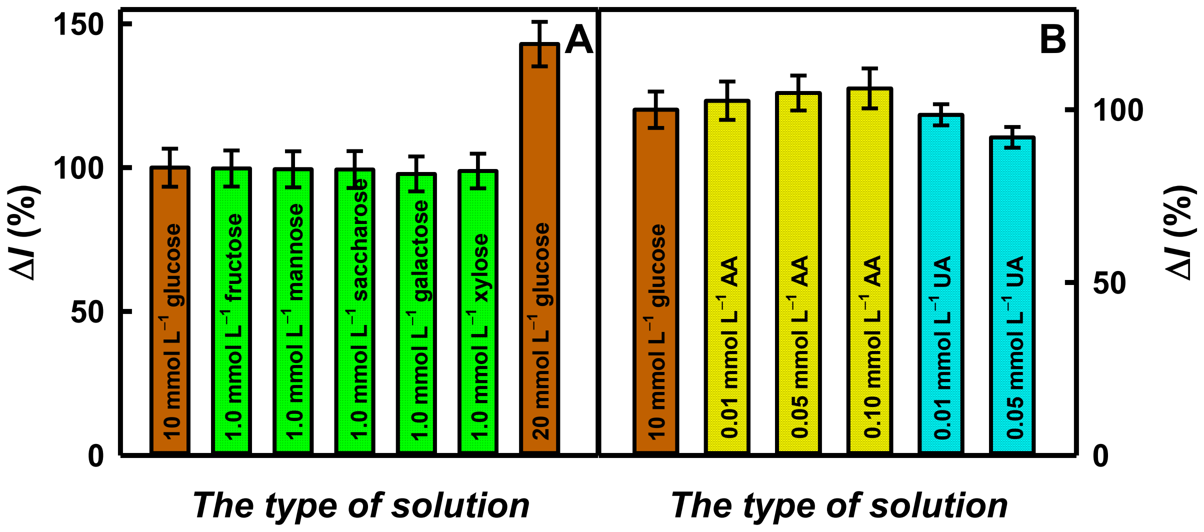

Figure S7. Influence of saccharides (A) and electroactive species (B) on the current of the hybrid G-EBFC based on the GR/AuNPs/Cys/PD/GOx bioanode. Details for presented plots: 10-fold diluted sample of human serum after the addition of A – 10 mmol L^{-1} of glucose (brown column), 1.0 mmol L^{-1} of saccharides (dotted green columns) and once more 10 mmol L^{-1} of glucose (brown column); B – 10 mmol L^{-1} of glucose without (brown column) and with AA (dotted yellow columns) or with UA (dotted azure columns). All measurements were performed using 49.0 kΩ of an external resistance.

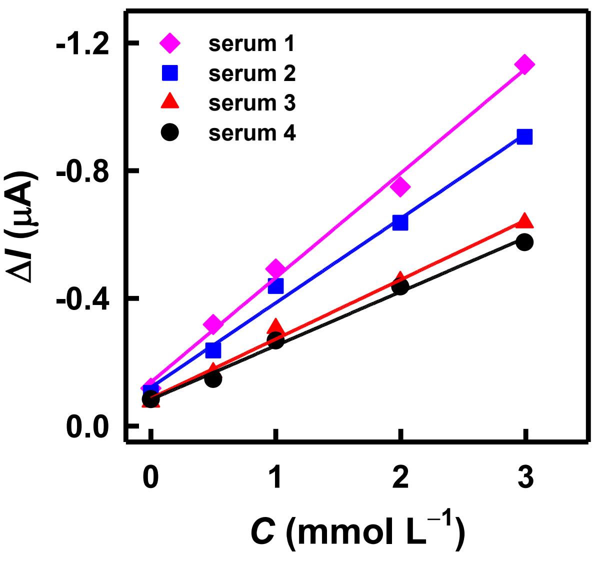

Figure S8. Detection of glucose in a 10-fold diluted sample of human serum, containing 0.484 mmol L^{-1} of glucose, by the 'standard addition' method. Measurements were performed using hybrid G-EBFC based on GR/AuNPs/Cys/PD/GOx bioanode at 49.0 kΩ of an external resistance.
